# Modern Technologies for Improving Broiler Production and Welfare: A Review

**DOI:** 10.3390/ani15040493

**Published:** 2025-02-10

**Authors:** Lili D. Brassó, István Komlósi, Zsófia Várszegi

**Affiliations:** Department of Animal Science, University of Debrecen, Böszörményi Street 138, 4032 Debrecen, Hungary; komlosi@agr.unideb.hu (I.K.); varszegi@agr.unideb.hu (Z.V.)

**Keywords:** PLF technologies, animal welfare, broiler behaviour, growth intensity, broiler production

## Abstract

The increasing level of poultry meat production, the lack of human workforce, and the rapid development of information technology have led to the application of precision livestock farming (PLF) systems in the poultry sector, as in other livestock sectors. This review aimed to gather information on the function, applicability, advantages, and limitations of a wide range of precision technologies applicable to broiler production to help farms and researchers in choosing the right methods in practice or creating the basis for further development. Studies as the basis of this review were extracted from more than a hundred peer-reviewed articles including mainly publications of recognised journals and conference proceedings. The results showed that most precision tools are currently undergoing testing and focus on some parameters or a group of parameters which are closely related. Information on the prevalence of PLF systems on broiler farms is not available in the literature. The accessible technologies of different purposes should be combined and connected to enable communication with each other and create a complex, reliable, and precise background for farming. This also facilitates management decisions and the treatment of so-called “Big Data”.

## Simple Summary

Precision livestock farming (PLF) has been at the forefront of research over the past decade and mainly in recent years. The growing demand for animal products and the lack of human workforce together with the improvements in digital technologies necessitates the development of reliable, continuous, and real-time systems based on artificial intelligence. Our review aimed to provide a concise overview of the advantages, possibilities, limitations, and disadvantages of available PLF technologies applicable to broiler farms. Unfortunately, most literature reports experimental data and only a few of them provide examples and experiences from the practice. However, based on the gathered knowledge and information, farms can decide whether to apply the presented tools and equipment, and through suggestions, we aimed to create possibilities for the further development of precision technologies.

## 1. Introduction

The global demand for chicken meat is increasing [1], necessitating improvements in production efficiency, meat quality, animal health, and welfare [2]. Modern farms aim to reduce production costs and labour needs while maintaining or increasing flock sizes, which has negatively impacted animal welfare and behaviour [3]. Alongside declining labour availability [4], these factors have driven the adoption of intelligent technologies [5]. Various PLF tools can help reduce production costs by monitoring environmental conditions, animal health, behaviour, and performance in real time [6,7,8,9]. PLF technologies are non-invasive, minimising stress on birds [10]. These technologies involve mechanical (sensors and robots) and digital (computer systems and software) tools, as well as socio-economic factors such as education and technology adoption [11]. Measuring feed and water consumption can help detect issues with feed quality and animal health more easily [12], while automated data collection can provide up-to-date information on production parameters and welfare indices, enabling early problem detection [8]. Precision technologies can also enhance decision-making processes in production management and reduce economic losses [13].

**Figure 1:**
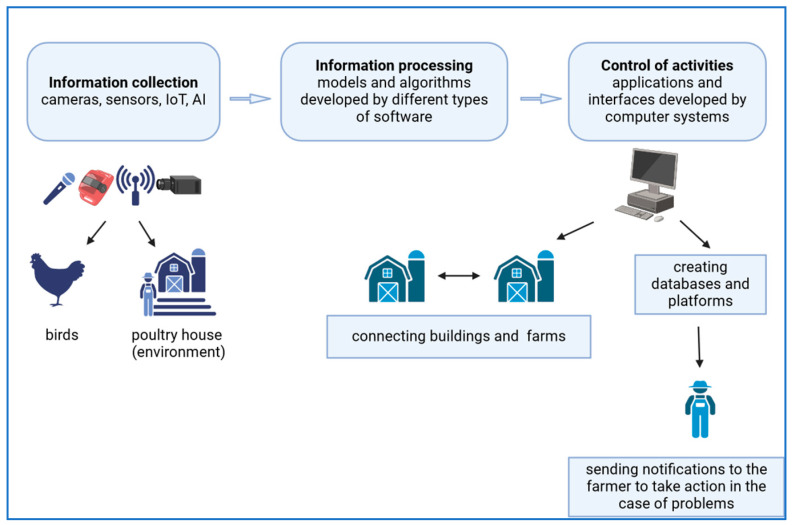
Schematic figure of information perception by PLF technologies. The figure was created with BioRender.com.

PLF systems involve three levels: information/data collection, processing, and supervision [14]. Information perception uses sensors, IoT (Internet of Things), and algorithms as accurate and automatic tools [14]. These systems, combined with artificial intelligence, support various areas of poultry production, including environmental conditions [15,16], animal behaviour [17,18], health [19,20], welfare [21], and production efficiency [22,23]. The process of data perception, transformation, and activity control in general is presented in Figure 1.

Numerous precision tools are available on the market, providing software, systems, networks, and devices for sound analysis, image and video analysis, weight and welfare monitoring, and the control of environmental conditions (air composition, light intensity, litter quality, temperature, etc.).

This review presents and critically reviews the available sources (application, advantages, and limitations) of information on a wide range of precision livestock farming technologies, including all the significant methods used in broiler production or in research to improve animal health and welfare, nutrition, and feed management practices.

## 2. Materials and Methods

### 2.1. Literature Search

The literature search was conducted using Google Scholar, ScienceDirect, Scopus, PubMed, and Web of Science databases between September 2023 and January 2025.

### 2.2. Searching Criteria

Searching criteria on all databases included keywords and phrases such as “precision technologies”, “PLF technologies”, “environmental conditions”, “sensors”, “RFID identification”, “machine learning”, “sound analysis”, “optical flow analysis”, “broiler growth”, “broiler welfare”, “image analysis”, “thermal imaging”, “feed monitoring”, and “limitations” in different combinations to gain as many results as possible. Furthermore, several articles cited manuscripts with topics under the scope of this study. The cited manuscripts were then searched in one of the above-mentioned databases. Filters for the year of publication were not used since we intended to access all the potential research materials according to the search criteria. Peer-reviewed manuscripts were the most preferred in the case of selection, but conference papers and theses were also accepted. However, websites and non-authorised, less recognised information sources were not included to provide the highest possible quality of review.

## 3. Precision Tools for the Control of Environmental Factors

Data collection on environmental parameters supports animal welfare, health, and production. Heat stress is a common problem in the case of broiler (meat-type) chickens with intensive metabolism and high growth rates [24]. The environmental temperature exceeding the birds’ thermoneutral zone hinders their heat release, leading to health problems, decreased production, and food safety [25]. When heat is unequally distributed inside the house, some areas may be colder. Chickens in those parts of the building gather to obtain warmth. Cold stress may weaken the immune system, cause digestive problems, and lead to a decrease in growth rates, negatively affecting productivity [26]. Each broiler house should be equipped with a ventilation, heating, and cooling system for controlling temperature, CO_2_, and ammonia levels and relative humidity [27]. Ventilation has a significant role in climate control inside livestock buildings maintaining the desired climatic conditions in every area of the houses [28]. The continuous monitoring of air characteristics, ambient temperature, relative humidity, ventilation intensity, light intensity, dust content, and CO_2_ and NH_3_ emissions inside buildings is crucial for maintaining animal health, preventing heat stress, and avoiding diseases [29]. Hygiene is another critical aspect of environmental conditions and disease prevention. Robotic systems, through artificial intelligence, collect data on birds’ immune status and spread litter enhancers, vaccines, and disinfectants [30,31]. Stocking densities are also regulated to ensure welfare [27]. High population densities and consumer awareness of animal feeding and housing conditions have led large-scale farms to adopt precision management tools to meet market and regulatory demands while maintaining production efficiency and profitability [32,33].

Many types of building simulation software available on the market use equations to control ventilation [28]. Sensors gather information on temperature, light intensity, air composition, and relative humidity and detect areas in the barn with inappropriate ventilation [34]. The setting of the ventilation system is possible through the analysis of sensor data by artificial intelligence (AI) algorithms [34]. Proper ventilation may lead to improved air quality, increased productivity, and reduced energy consumption [35]. As well-known machine learning tools, computational fluid dynamics (CFD) can be used for the real-time or model-based prediction of airflow in livestock houses by controlling the heating and cooling systems and the mechanical ventilation through windows. The operation of the model may be challenging in the case of large flock sizes (thousands of animals) and a high number of operable windows [28]. Sensors and sonic anemometers provide autonomous two-dimensional data collection on climate conditions, air composition, bird distribution, and activity [15,16]. IoT (Internet of Things) may be a promising tool for the real-time, reliable, and cost-effective monitoring of environmental data including temperature, relative humidity, ammonia, and light intensity. The devices provide accuracies above 91% and a 77.34% lower cost compared to digital equipment [36]. The system is based on sensors, software, computers, interfaces, and the internet. IoT technologies can connect sensors, devices, and equipment and enable the automation of tasks within a farm [37]. Machine learning (ML) for the control of environmental conditions (i.e., air composition and lighting) comprehend a Multi-Input Multi-Output (MIMO) fuzzy controller combined with a proportional, integral, and derivative (PID) controller (MFLPID) [38]; neuro-fuzzy and neural network techniques [39]; and deep learning [40]. Under experimental conditions, the MIMO fuzzy controller could reduce energy consumption by 43% compared to the ON/Off system, providing high accuracies (3% maximum error for temperature and 4% maximum error for relative humidity). The neural network has several types, such as multilayer perceptron (MP), the adaptive neuro-fuzzy interference system (ANFIS), grid partitioning (GP), subtractive clustering (SC), and multiple linear regression analysis (MLR) using linear equations [39]. In a study, a neural network was used for the detection of ammonia concentration in a broiler house. The ANFIS-SC model provided the highest accuracy (R^2^ = 0.858) followed by the ANFIS-GP (R^2^ = 0.853), MP (R^2^ = 0.837), and MLR (R^2^ = 0.667) for ammonia estimation based on litter temperature, humidity, and pH input data. Multipurpose robots can measure air composition, light intensity, noise level, and litter quality [16] and sanitise broiler houses [41] while turning and aerating the litter to reduce pathogen numbers and foot problems [16]. The addressed technologies can have great potential to be applied in practice; however, most of them are on the level of testing only on a few farms.

## 4. Precision Tools for Assessing the Behaviour, Welfare, and Health of Birds

### 4.1. Sound Analysis

Animals use sounds to communicate their physical and emotional states, establish social relationships, and signal stress. Chickens can emit 30 types of sounds [42], with vocalisations indicating readiness for breeding, pain, stress, or need [19]. Changes in vocalisation often reflect physiological and behavioural changes [43], with most signals showing certain patterns that express the motivation of the individuals [44]. For example, tonal sounds are associated with fear, while harsh ones indicate aggression [45]. Vocalisations vary depending on the threats’ origin and birds’ motivations [46]. In the case of food reward, chickens can express calls with different frequencies and rates based on the food type [47]. In this respect, animal sound as a form of communication carries information encoded in the amplitude, frequency, duration, rate, and energy distribution of the voice [48,49,50,51,52]. The vocalisation of animals in response to environmental stimuli depends on both the type of environmental effects and the individuals’ perception (i.e., inner emotional state) [42]. Sound analysis may be used in poultry systems to assess welfare and health [42] and may also be applied for day-old sex and genetic strain identification [53]. The development of sound-based systems may be a promising tool for detecting the symptoms of avian influenza or other respiratory diseases [54]. According to Banakar et al. [55], the amplitude of birds’ audio signals differs in the case of healthy individuals and birds infected by Newcastle disease, the bronchitis virus, and avian influenza. The authors conducted research on Ross 308 broiler chickens to detect the above-listed diseases using a Support Vector Machine (SVM) classifier, data-mining methods, and Dempster–Shafer evidence theory (D-S). The transformation of acoustic signals to frequency and time-frequency domains was performed with Fast Fourier Transform (FFT) and Discrete Wavelet Transform (DWT). The results showed 41.35% accuracy for SVM-based detection and 91.15% accuracy based on the D-S evidence theory infusion. In their study, Carpentier et al. [54] applied a SOMO+ device for audio recording, Audacity^®^ recording and editing software for labelling, and algorithms were performed in MATLAB (The Mathworks Inc., Natick, MA, USA, 2017). After filtration, sneeze and no-sneeze sounds could be classified with 66.7% sensitivity and 88.4% accuracy. Bioacoustics can be used to perceive and evaluate coughs in a real-time, non-invasive manner by their resonance [56]. In an experiment, Mahdavian et al. [57] used sound analysis for the detection of the Newcastle virus and bronchitis in broiler chickens based on their vocal signals using an acoustic box, Mel-frequency cepstral coefficient acoustic features, wavelet entropy, the MFCCs calculation algorithm, and a Support Vector Machine (SVM) classifier. Wavelet entropy showed the highest accuracy of 83% in the case of bronchitis on day 3 after inoculation. Regarding Newcastle disease, the Mel-cepstral coefficient and wavelet entropy presented 78% and 80% accuracies in differentiating healthy and unhealthy birds. The listed, mentioned studies highlight that sound analysis may have a great potential for the improvement in broiler chicken health and welfare. However, methods and technologies are mainly under testing and used in experiments rather than in practice and require further assessments regarding their applicability on farms.

Neural network pattern recognition can identify chickens infected by *Clostridium perfringens* type A with accuracies between 66.6% and 100% [19]. The neural network differentiates between healthy and unhealthy chickens based on acoustic signals, with feature extraction supporting that healthy individuals show more intensive and uniform vocalisation compared to unhealthy ones [19]. A sound analysis of broiler chicks by noise frequency and amplitude spectrums using audio editing software informs us about birds’ thermal conditions and well-being [58]. A higher vocalisation frequency was associated with chicks flocking together, indicating a lower-than-optimal temperature, while an increased vocalisation amplitude was related to the normal distribution of chicks within the poultry house, showing that the temperature was within the comfort range of birds [58]. Vocalisation frequency changes during incubation stages are detectable by electret microphones and algorithms [59]. Using an electret microphone and an algorithm developed by audio software based on the sound of the developing chicken embryos, it is possible to determine the time of internal pipping. The algorithm detects when 93–98% of chicks enter the internal pipping stage, enabling the reduction in the hatching window and resulting in lower chick mortality [59].

### 4.2. Image and Video Analysis

The intensive growth of chickens leads to a decrease in locomotion and physical activities [60], with reduced activity frequently being a sign of foot problems (i.e., foot pad dermatitis and hock burn) caused by fast growth and poor litter quality, posing a common problem in broiler production [61,62]. The ulcers are painful and decrease locomotor abilities, reducing feed and water intake, and resulting in weight loss [63].

#### 4.2.1. Three-Dimensional Vision Monitoring, Deep Learning, and Neural Networks

Artificial neural networks, machine vision, and 3D computer vision can be used for the weight monitoring of broiler chickens based on image analysis. Amraei et al. [64] applied machine vision and artificial neural networks for the estimation of broiler chicken body weight from 1 to 42 days of age. Images were taken two times daily, and weight estimation was performed by the Levenberg–Marquardt, gradient descent, Bayesian regulation, and scaled conjugate gradient algorithms. The Bayesian regulation algorithm was proved to have the highest reliability (R = 0.98). However, according to Peng, the method provides only approximate measurements with errors around 50 g. Also, small changes are difficult to track in real time, and bird identification and the determination of feed intake simultaneously are technically challenging [65].

A 3D vision monitoring system supplemented with algorithms may be an essential, non-invasive, and non-intrusive tool for broiler gait scores and lameness assessments, providing 93% accuracy [17]. RFIDs (Radio Frequency Identification Devices) tags fixed on the back of chickens in a backpack help to track the locomotion and behavioural activity of birds [66], although the expensive nature and sensor inaccuracy of these devices imply a need for further development [12].

Deep neural networks such as the single-shot MultiBox detector (SSD), feature fusion single-shot MultiBox detector (FFSSD), or their improved versions with classification models of InceptionV3 and VGG16 can determine the health status of birds [67], although their disadvantage is the limitation in the number of animals per image and the image size. Digital image processing methods and several deep learning algorithms (i.e., R-FCN, R-CNN (constructed convolutional neural network), YOLO-V3, etc.) can help to detect the health status of broilers [68,69], with accuracies between 84% and 100%. However, due to the high similarity of the shapes of healthy and dead chickens, the recognition of dead ones is challenging and may affect the precision of classification [70]. Collins [71] investigated the correlations between stocking densities, social relationships, and the behaviour of broiler chickens using video and path analyses to track the birds’ movements. His findings indicated that stocking density had a significant effect on individual behaviour.

#### 4.2.2. Optical Flow Analysis and Linear Models

Optical flow analysis enables the continuous evaluation of bird locomotion by assessing the velocity of change in the brightness of image pixels, indicating leg problems without further tools and sensors [72]. Based on camera recordings, optical flow combined with the Bayesian regression model is a great tool for mortality, weight gain, foot pad dermatitis, and hock burn estimation [73]. Dawkins et al. [72] established a negative correlation between the mean optical flow and the mortality of birds, also declaring that the skew and kurtosis of optical flow were positively correlated with welfare indices such as mortality (r = 0.42; r = 0.45), gait scores (r = 0.42; r = 0.48), and hock burns (r = 0.57; r = 0.56). Solid predictions were carried out as early as day 15 of life [72]. Higher optical flow values were associated with increased activity (r_sitting_ = −0.21; r_walking_ = 0.42). The optical flow analysis also indicated that the variance in the chickens’ walking ability was lower in the flock with more uniform movements. It means that the uniformity in the movements of the examined flock indicated a better walking ability and hence better foot health. The accuracy of the technology was not provided in the literature [74]. The optical flow analysis can also include activity, distribution, and activity/distribution indices [75]. The basis of the calculations is a camera image of chickens containing segments and grids. To obtain zone occupation density as a distribution indicator, the areas of the segmented chickens and the grid are compared. In the case of the activity index, the regions of active and segmented chickens are compared [69]. The activity/distribution index is calculated by dividing the size of zones having average flock activity within a range of 25% with the zone number. Strong correlations were established between the predicted and observed gait scores based on the optical data, ranging between 0.85 and 0.97 [74]. This technique provides up-to-date information on bird health (i.e., gait scores) at an early age, before the appearance of symptoms in a cost- and labour-effective manner [72,73,74]. The algorithm applied in this study was an initial form of individual gait score assessment by precision methods, implying a need for further development and validation.

In another study, a remote monitoring system operating with three cameras provided continuous data collection on population densities, bird activity, and distribution analysis. The system used zones and pixels for activity and distribution index calculations [18]. The behaviour and welfare of birds may be well estimated by this method, focusing especially on the ratio of chickens visiting the feeders and drinkers [18]. The system seemed appropriate to also determine the correlation between the activity and lameness of broiler chickens based on the gait scores [2]. However, it should be taken into account that correct definitions should be provided for data classification (i.e., eating or drinking birds) for the right and precise application of the model [18]. The environment of the observed zone should also be analysed, because in specific areas, birds can be without feed intake [18]. Another method is the use of force plates that can inform the farmer about the gait health of broilers [76]; however, the results are questionable, inaccurate, and statistically insignificant in many cases. For example, in the experiment of Corr et al. [76], birds with higher growth rates had lower speeds and thus ground reaction force; however, it was not related to lameness or other gait problems. In the study of Silvera et al. [2], for the prediction of gait score measures, the baseline activity, amplitude (moving away from the observer), time to return to baseline activities, and average activity after were used as variables. The amplitude was related to the walking ability, and it was also reliable for the prediction of the relationship between humans and chickens [2]. Finally, dynamic linear models (DLMs) based on video image analysis seem to be suitable for the evaluation of broiler chicken activity at a given age and life period [77]. Variations and deviations from normal activity (outliers) may be detected by this automatic monitoring system using filters. Thus, the farmer can be notified to take action in the case of unexpected problems (i.e., sudden, detrimental changes in the temperature or relative humidity) to improve animal welfare [78].

#### 4.2.3. Thermal Imaging

Thermal imaging can help in behaviour analyses in bird flocks, providing time-saving, objective, and continuous methods that may be well applied in foot health investigations [16], and it is also a useful way to optimise the heating and ventilation of broiler houses [79]. There are two main methods for measuring body temperature: contact and non-contact [80]. Contact devices may cause stress to the birds and do not support the concept of animal welfare [81], so these technologies are not mentioned in the manuscript. Non-contact methods, such as infrared thermal imaging cameras, are non-invasive techniques that can measure body surface temperature and are frequently applied in the identification of pathological changes and symptoms [81,82]. Infrared cameras used in thermal imaging measure the infrared radiation (wavelengths) of a surface or the abdominal skin temperature of a broiler chicken [83,84]. The generated thermal map can be used for the early detection and prediction of infections [85] and plays a significant role in the physiological and diagnostic research of mammals, birds, and humans [86,87]. Noh et al. [88] used an SM080TIP thermal camera and Argus viewer software program for the detection of the highly pathogenic avian influenza virus in broiler chickens and ducks. The authors found a positive relationship (*p* < 0.05) between maximum body surface temperature and virus infection, peaking at 42.9 °C 24 h post-infection. Sadeghi et al. [89] conducted research on boiler chickens with thermal imaging to detect the presence of Newcastle disease and avian influenza. The system was based on thermal cameras, artificial neural networks, and Support Vector Machines as classifiers and FLIR and MATLAB software for image processing. According to their findings, the Support-Vector-Machine-based system achieved 97.2% and 100% accuracies for avian influenza and Newcastle disease classification within 24 h after infection. Nääs et al. [35] used thermal imaging technology to estimate thermal comfort in broiler chickens. By applying video recordings taken in a climatic chamber and algorithms, the analysis of behaviour may serve as a significant welfare indicator in response to the climatic environment. Thermal cameras of ChickenBoy robots, combined with artificial intelligence, can measure the composition of faeces for the detection of diarrhoea. Data can be uploaded to a cloud and extracted to the computer or forwarded to smartphones. The inbuilt maps can help to direct the farmer to problematic areas by providing notification sounds and signs [20]. The robot can detect dead birds by distinguishing between live and dead individuals and scanning wet parts in the litter using infrared thermal imaging [16]. The accuracy and effectiveness of its activity are not mentioned in the literature. According to Lu et al. [82], thermal imaging is rarely used for poultry in general due to their small body size and feathers.

## 5. Tools for the Precision Feeding and Growth Estimation of Broiler Chickens

### 5.1. The Monitoring of Water and Feed Intake

Feed constitutes 70–80% of poultry production costs, making real-time control of inputs crucial [84]. Precision feeding can include the accurate measurement and distribution of the delivered feed quantity adjusted to the age and breeding purpose of birds. The time and frequency of feeding can also be determined but mainly in the case of broiler breeders. Feed intake and the feed conversion ratio can be calculated as well [90]. Precision feeding may enable uniform flock body weights and increase cost efficiency [91].

The control of feed and water consumption in broiler houses is based on electronic load-cell scales and flow meters fixed on water pipes and troughs. Load cell scales and water meters in each row or the whole house are used to estimate feed and water consumption. These simple tools help detect the health status of birds and any technological and feed quality problems [92,93]. However, many studies revealed the possible applicability of RFID systems as novel technologies among experimental circumstances for the monitoring of individual feeding and drinking behaviour in poultry and pigs [94,95,96,97,98,99]. The accuracy of the system for broiler chickens was reported between 92.5 and 99% [96]. An optical flow analysis combined with the Bayesian regression model may also be well applicable for the estimation of the water and feed consumption of birds [73]. Problems with the operation of feeder lines can be controlled by computer vision based on the distribution index of broiler chickens [100]. A lower distribution index is observed in the case of limited access to the feeder. The accuracy of the system is 95.24%, expressing a high reliability. There is no contradiction mentioned in the literature questioning the usability of the technology [101]. Metabolic robots can be effective tools in improving the FCR (Food Conversion Ratio) of birds by 4% by adjusting feed doses according to the bird’s specific needs at a given age or development phase and controlling feeding time [102]. The robots can be operated by solar energy and are wireless, easily installable, self-monitoring, and provide continuous, real-time information about the amount of feed stored in the silos, enabling the control of feed intake [12]. Opportunities and challenges are not available. The individual feed intake of broilers can be measured in vitro by sound analysis [103]. Feed quantity is estimated by a weighing system with a balance placed underneath the feeder. Video image recordings with webcams are used to measure pecking frequency. Electret microphones attached to the trough are used for the detection of pecking noise and the preparation of the pecking algorithm. Algorithms are validated by computer vision technology, with an accuracy of 93% [104].

### 5.2. The Monitoring of the Growth Rate

The weight gain of birds indicates the economic efficiency of farming. Data collection on chicken body weight may inform us about the uniformity of chickens in growth intensity, body weight, feed conversion ratio, and health condition [21]. According to Wang et al. [22], the individual monitoring of broiler chickens’ growth can be conducted based on RFID identification and electronic weighing. However, the authors note that the disadvantage of this system is the position of the tags on the chicken’s body, which causes stress for them, resulting in unreliable results [22]. In general practice, growth models are used for the estimation of the growth rate and nutrient requirements of chickens considering feed composition and environmental conditions [104]. Allometric growth equations can also be calculated [105], and feed formulators and economic optimisers can be implemented in the model, enabling economic feed formulation [104]. Apart from growth models, a precision feeding system was developed recently in Canada for broiler breeders. In this case, a feeding station is applied for individual feed delivery depending on the difference between the real-time and target body weight of birds [92]. The future system will enable nutrient specifications based on the individual needs of breeders [92]. A novel way of weight monitoring in broiler chickens is a system based on the analysis of perching behaviour. Wang et al. [22] investigated the accuracy of an image-assisted rod platform weighing system based on a strain-gauged sensor for the determination of broiler chickens’ weight. The automated weighing system provided accuracies within 2% for average weight and 1.5% for flock uniformity, which is more precise compared to manual ones of 0–5% [22].

Several authors revealed associations between sound frequency and the weight of birds as an innovative approach to weight monitoring [23,106]. Fontana et al. [23] investigated the accuracy of weight analysis by the sound of birds and established a strong correlation (r = 0.96) between the expected and observed weights. Their findings were consistent with that of Bowling et al. [106], who found an inverse relationship between the body size and vocalisation frequency of birds during the production cycle. Higher frequencies at younger ages indicated lower weights and vice versa [106]. No study has questioned the accuracy of sound analysis, so it can be a reliable method of weight determination. Initial forms of image analysis first applied to estimate live weight and carcass characteristics in swine date back to the 1980s–1990s [107]. These techniques provided more reliable measurements than manual ones [9]. De Wet et al. [21] were the first to report on the use of image analysis systems in broiler production. In their study, the body surface area and dimensions captured from a top view were the basis for determining chicken live weight. A Sobel–Feldman operator was used for image processing, and regression analysis was applied to establish the relationships between body dimensions and live weights [21]. Results showed the reliability of the method to be lower (89%) compared to the same analysis conducted with pigs (95%). The locomotion of chickens resulted in alterations in their orientation and posture, leading to inconsistent variations between individuals [21]. Mollah et al. [108] used the body contour of birds for weight monitoring. Measurements were based on a digital camera placed 1 m high above the bird to be captured. With optimal light intensity, its body contour became easily visible and was displayed as the average of ten image replicates. The number of pixels in the body surface area was calculated by image processing software, and data were used in the estimation of body weight. Estimation errors ranged between 0.04% and 16.47% regarding surface area pixels. Up to 35 days of age, calculations did not differ from the manual observations (*p* > 0.05). However, according to the authors, the system needs development to improve its practical application [108] Table 1. A novel method of broiler weight determination is dynamic weighing [59]. The system provides individual monitoring of feed intake and growth by integrating precision feeding and weighing technology, an RFID reading module, analogue circuits, digital filtering, and calculation equations. However, the accuracy is only 2.16 g in contrast to the 0.82 g weighing accuracy of static weighing [59].

The use of light as a point laser to raise the curiosity of chickens may improve bird activity and feed consumption, resulting in higher growth rates [109].

## 6. Limitations of PLF Technologies

Before applying precision technologies, we should be aware of possible problems and limitations that may arise during the operation of systems and devices. This may help us in planning, decision making, and problem solving.

According to Morrone [110], we should face an almost equal number of advantages and disadvantages of PLF systems. Technical failures and malfunctioning may emerge caused by power cuts, hardware breakdowns, tag losses, etc. [111]. It can be a great challenge for farms that are highly dependent on PLF tools because in most cases, they are unable to solve the problems on their own and need to ask for specialists [111]. Contact devices (i.e., tags) tied or stuck on the body may negatively affect animal behaviour and disturb animals, causing stress for them (i.e., location tracking in boiler chickens or a body-surface-temperature-measuring gauge and implantable radio-telemetry systems for the estimation of heart rate and deep body temperature in laying hens) [12,90]. Also, they may pose a threat to pathogen transmission that may arise by the inappropriate disinfection methods of robots (i.e., automatic milking in dairy cows) [112]. Algorithms may provide less reliable results under real farm conditions compared to in vitro analyses. This is mainly due to the poor quality and low variability of training data [113]. For example, in the case of behaviour analysis by machine vision, the technology may present great advancements under experimental conditions and later may require a further, on-farm validation of its performance. Also, the testing of machines and devices on young animals (i.e., a collar or accelerometer on calves) may need a more detailed assessment before the application [114]. Many tools have been applied only under experimental conditions, but they could also be used in commercial circumstances [12]. However, most PLF technologies (i.e., 14% of sensors in dairy cattle and 23% of PLF in pigs) are under validation, which should be a complex process considering as many factors as possible (i.e., the individuality and timely variability of organisms, etc.) [115]. Often, there is no correspondence and agreement on the intentions of researchers, developers, and manufacturers in the scope of indicators and parameters to be measured when assessing the applicability of PLF tools (i.e., a lack of information on production efficiency) [114]. Under- or over-reliance on PLF technology can also pose a threat to animal welfare and farm productivity, mainly in the case of failures on entirely automated farms, which is often caused by the lack of knowledge of users on the usability and limitations of PLF devices [112]. PLF systems may result in a decrease in human–animal contact, and interactions can be mainly limited to the less favourable ones (i.e., vaccination, transportation, etc.), which pose stress to the animals [115]. The application of PLF tools may reduce the need for human skills and efforts (i.e., in identifying alterations from normal behaviour or health issues) to take care of the animals, risking the ability to notice problems and take action in the case of PLF system failures [112]. Regarding farm levels, PLF tools cannot be profitably and easily operated on small-scale, extensive farms due to the poor technological background, too large areas to be covered, and the high production costs per animal [90,116]. Also, the expenses of these technologies (sensors, devices, automated feeding and milking systems, GPS, Wi-Fi network, power supplies, etc.) usually take a long time to be paid off [16,117]. Currently, the focus of PLF systems is more on production efficiency and economic aspects than on animal welfare, which may diminish the moral status of animals in society [118].

There is scarce information on the operational success and effectiveness of robots. This is the consequence of the limited number of experimental studies in this field and also because designer commercial companies are not willing to disclose data on these technologies [64]. Moreover, poultry robots are limited in functionality and applicability because most of them are designed for specific tasks (i.e., egg collection, bedding, feeding, litter sanitisation, chicken harvest, etc.) [36]. Multi-tasking abilities (i.e., the combination of the available tools and devices mentioned in the review) should be expanded by using advanced technologies and AI by facing various environmental conditions during navigation (i.e., manoeuvrability, stability, controllability, etc.) in the poultry house [119]. Also, there is a need to develop an obstacle awareness system to improve real-time path planning and navigation [64]. The social learning abilities of robots to improve and facilitate robot–animal interactions and future research objectives should focus on this field of challenges regarding animal welfare [120].

## 7. Future Directions for Overcoming Limitations

Based on their several advantages, PLF technologies (providing valuable, real-time information on environmental conditions, animal physiology, health, welfare, production, and location and facilitating the decision making of farmers) can contribute to lean management, sustainability, and resilience as main principles in today’s economy and farming. Climate change is known to adversely affect animals’ health and production [121,122]. Overcoming and the prevention of its effects will be a significant future challenge. Precision tools can provide promising strategies for alleviating heat stress by continuously measuring the physiological parameters of the animals or the optimisation of feed intake to reduce metabolic heat production [10]. Precision nutrition can alleviate the negative effects of heat stress and contribute to the improvement of animal health, feed efficiency, uniform growth, meat quality, and hence the sustainability of production [10]. Stress and health problems can be monitored and detected at an early stage and hence can be treated, mitigating suffering and expenses on treatments and production losses [123]. Precision technologies can incorporate business management software platforms to enable the integration of other business operations including finance, regulations, and inventions [124]. Portable devices, artificial intelligence (AI), and Internet of Things (IoT) are gaining increasing significance and attention, so web-based, remote monitoring of facilities using mobile applications may be the future of livestock farming [125].

## 8. Conclusions

The application of precision technology in agriculture and the poultry sector dates back to the 1980s when the first forms of digital image processing were initiated. Nowadays, with the rapid growth of information and computer technology, the scope and type of PLF tools are increasing. Our review presented the available sources of information on a wide range of PLF technologies applicable to poultry production. Sensors can provide continuous and real-time data on environmental conditions (i.e., temperature, humidity, ventilation speed, CO_2_ and NH_3_ concentrations, etc.) that are forwarded to a system. Extracted data can be used for control, and the farmer is alarmed in the case of problems to interfere with. Three-dimensional vision monitoring and digital image processing methods with deep learning algorithms can detect lameness and diseases with high accuracies (from 84% to 100%). Multipurpose robots can measure many parameters in parallel, which can be energy and economic friendly and help to have a more exact view of production and welfare.

According to the available literature sources, despite their advantages, the application of PLF tools and technologies is reduced mainly to the experimental level. However, as studies revealed, they could be used under commercial circumstances after validation. Reasons that commercial farms are reluctant to apply PLF technologies include the lack of validation of technologies, limited capacities in the case of large flocks and small body sizes (i.e., chickens), high investment costs of devices (i.e., RFID and robots) especially on small-scale farms, the lack of knowledge of users (i.e., farmers, caretakers, etc.), the possibility of technical problems and need for special services, and the specificity of tasks of different devices [12,36,111]. Regarding the mentioned limitations, the following suggestions can be made for the future investigation and improvement of PLF technologies. Globalisation and the increasing need for animal products desire the integration of PLF and IoT technologies (i.e., smartphones; fixed, environmental, and wearable devices equipped with sensors; accelerometers; and cameras for behaviour and health monitoring and location tracking) [125]. Based on artificial intelligence, the operation of sensors, robots, and other systems should be synchronised, leading to more effective data collection and processes providing a concise overview of farm productivity. Tools should be connected to enable communication with each other and create a complex, reliable, and precise background for farming. This also facilitates management decisions and the treatment of so-called “Big Data” [126]. When designing and constructing PLF equipment, the ethics of sustainability should be considered as well [127]. Considering the above-mentioned perspectives and possible objectives, the development of future precision livestock farms may be promoted hand in hand with the improvement in animal welfare and sustainability.

## Figures and Tables

**Table 1:** Summary of precision broiler technology tools with applications, advantages and limitations.

Aim of Application	PLF Tool/System/Network	Advantage	Reference
Real-time or model-based detection of air conditions and light intensity	Computational fluid dynamics (CFD)	Cost-effective and time-saving compared to traditional study methods	[28,39]
	Machine learning (MIMO, ANFIS-SC, ANFIS-GP, MP, MLR, MFLPID, FLC)	Highest accuracy for ANFIS-SC of R^2^ = 0.86 R^2^ for MFLPID = 0.97 14 to 43% energy saving for MFLPID	[37,38]
2-dimensional data collection on climate conditions, air composition, bird distribution, and activity Information on gait score and hock burn	Sensors and ultrasonic anemometer (USA)	Low cost, fine temporal resolution (20 Hz)	[15,16]
	Optical flow analysis	Solid prediction of mortality, gait score and foot health Provides the assessment of several hundreds of animals together automatically and continuously	[72]
Robots for monitoring of air conditions, turning and sanitisation of the litter, dead birds, water dripping	Octopus Poultry Safe^®^, ChickenBoy^®^, Scout^TM^, Robochick^®^	Work in the presence of birds Reduce moisture and pathogenic bacteria count Cost-effective, continuous information on environmental conditions and bird health	[41]
			[16,20]
Robots for increasing bird movements	T-moov^®^, Spoutnic NAV^®^, AviSense Robot	Decreased escape distance	[15,70]
Robots for the reduction of stress and fear	Mobile robot, Ground robot, Mobile Robotic Prototype, Robotic Vehicle		[90]
Early disease detection, monitoring of deep body temperature	Sound analysis (WSN)	Long battery life (2 years)	[58]
Image analysis for health monitoring	RetinaNet^®^, R-FCN, Faster R-CNN, YOLO-V3	High accuracies between 84% and 100%	[23,68]
Precision feeding	Kai-Zen Feeding Robot^®^	Improvement of feed conversion rate (FCR) by 4%	[102]
	Feed Cast^®^	Self-sufficient and solar energy -powered, effective monitoring of feed level in silos	[96]
Detection of lameness and population densities Location tracking	EthnoVision^®^, TrackLab^®^, EyeNamic^®^	Accuracy of 95.24%	[19]
	3D-vision monitoring system	Accuracy of 93% for the detection of lying events	[60]
Weight monitoring	Sound analysis (Adobe^®^ Audition™)	Accuracy of R^2^ = 0.98	[24]
	Image analysis (IDRISI 32, MATLAB, LIBSVM)	Accuracy of R^2^ = 0.98–0.99	[108]
	Machine learning	Accuracies between 98–100%	[38]

## Data Availability

No new data were created or analyzed in this study.
